# Improving the estimation of alpine grassland fractional vegetation cover using optimized algorithms and multi-dimensional features

**DOI:** 10.1186/s13007-021-00796-5

**Published:** 2021-09-17

**Authors:** Xingchen Lin, Jianjun Chen, Peiqing Lou, Shuhua Yi, Yu Qin, Haotian You, Xiaowen Han

**Affiliations:** 1 College of Geomatics and Geoinformation, Guilin University of Technology, No.12 Jiangan Street, Guilin, 541006 China; 2 Guangxi Key Laboratory of Spatial Information and Geomatics, Guilin University of Technology, 12 Jiangan Road, Guilin, 541004 China; 3 Institute of Fragile Ecosystem and Environment, Nantong University, 999 Tongjing Road, Nantong, 226007 China; 4 State Key Laboratory of Cryospheric Science, Northwest Institute of Eco-Environment and Resources, Chinese Academy of Sciences, 320 Donggang West Road, Lanzhou, 730000 China

**Keywords:** Fractional vegetation cover (FVC), Alpine grassland, Unmanned aerial vehicle (UAV) aerial imagery, Machine learning algorithms, Feature selection, Parameter tuning, Accuracy evaluation

## Abstract

**Background:**

Fractional vegetation cover (FVC) is an important basic parameter for the quantitative monitoring of the alpine grassland ecosystem on the Qinghai-Tibetan Plateau. Based on unmanned aerial vehicle (UAV) acquisition of measured data and matching it with satellite remote sensing images at the pixel scale, the proper selection of driving data and inversion algorithms can be determined and is crucial for generating high-precision alpine grassland FVC products.

**Methods:**

This study presents estimations of alpine grassland FVC using optimized algorithms and multi-dimensional features. The multi-dimensional feature set (using original spectral bands, 22 vegetation indices, and topographical factors) was constructed from many sources of information, then the optimal feature subset was determined based on different feature selection algorithms as the driving data for optimized machine learning algorithms. Finally, the inversion accuracy, sensitivity to sample size, and computational efficiency of the four machine learning algorithms were evaluated.

**Results:**

(1) The random forest (RF) algorithm (R^2^: 0.861, RMSE: 9.5%) performed the best for FVC inversion among the four machine learning algorithms driven by the four typical vegetation indices. (2) Compared with the four typical vegetation indices, using multi-dimensional feature sets as driving data obviously improved the FVC inversion accuracy of the four machine learning algorithms (R^2^ of the RF algorithm increased to 0.890). (3) Among the three variable selection algorithms (Boruta, sequential forward selection [SFS], and permutation importance-recursive feature elimination [PI-RFE]), the constructed PI-RFE feature selection algorithm had the best dimensionality reduction effect on the multi-dimensional feature set. (4) The hyper-parameter optimization of the machine learning algorithms and feature selection of the multi-dimensional feature set further improved FVC inversion accuracy (R^2^: 0.917 and RMSE: 7.9% in the optimized RF algorithm).

**Conclusion:**

This study provides a highly precise, optimized algorithm with an optimal multi-dimensional feature set for FVC inversion, which is vital for the quantitative monitoring of the ecological environment of alpine grassland.

## Introduction

Known as the “Third Pole” and “Water Tower of Asia”, the Qinghai-Tibet Plateau (QTP) plays a very important role in regulating climate and water resources in East Asia and is thus regarded as the trigger and amplifier of climate change in Asia and even the Northern Hemisphere [76, 77]. As the main vegetation type on the QTP, alpine grassland has experienced serious degradation in the past few decades by the combined impact of climate warming, overgrazing, and rodent disturbance [17, 73]. Fractional vegetation cover (FVC) is an ideal indicator for the dynamic monitoring of the vegetation condition of the alpine ecosystems on the QTP [60, 61, 33, 48]. Therefore, high-precision FVC assessment of the alpine grassland on the QTP is of great significance as it provides insight into ecological environment changes and their accompanied influences [54, 82, 91].

Remote sensing technology has been widely used in FVC inversion at the regional scale. The inversion methods are generally divided into three categories: the regression model, the pixel dichotomy model, and machine learning algorithms. The regression model inverts FVC based on the statistical relationship between the vegetation index and measured data. Although this method is easy to implement, it is difficult to extend to other regions, owing to the limitations of the established model itself [25, 66]. The pixel dichotomy model generally determines FVC by dividing the surface features in the mixed pixel into vegetation and non-vegetation categories. However, it is difficult to find pure spectral pixels due to the restriction of the spatial resolution of remote sensing images [24, 38]; [79, 87]. Machine learning algorithms include multiple linear regression (MLR), back-propagation neural networks (BPNNs), support vector regression (SVR), random forest (RF), and a series of other algorithms [26, 39, 53]. The basic idea of machine learning algorithms is to invert FVC by simulating the intrinsic relationship between remote sensing information and FVC [72]. Although many types of algorithms exist, it is still unknown which has the best inversion accuracy and computational efficiency for FVC inversion.

In addition to the algorithm, the selection of features from the remote sensing dataset also has a great impact on the FVC inversion accuracy, such as vegetation indices calculated from original spectral bands of the remote sensing data [23]. The normalized difference vegetation index (NDVI), enhanced vegetation index (EVI), soil-adjusted vegetation index (SAVI), modified soil-adjusted vegetation index (MSAVI), etc., are usually used [83]. To date, it is unknown whether other vegetation indices have a higher correlation with the FVC of alpine grassland than typical vegetation indices. Given the obvious differences in the elevation of the QTP [92], there are great variations in the digital elevation model (DEM), slope, and aspect of the alpine grassland. The influence of these topographical factors, however, is neglected. Although it is considered that analysis driven by a multi-dimensional feature set including original spectral bands, various vegetation indices, and topographical factors can improve the FVC inversion accuracy of the machine learning algorithm, this still needs to be further explored as there are too many features in dataset and data redundancy will inevitably occur, leading to longer training time for the inversion model and overfitting [74, 88]. Therefore, it is essential to eliminate the redundancy of the multi-dimensional feature set, which helps to improve the inversion accuracy and calculation efficiency of alpine grassland FVC.

No matter which kind of FVC remote sensing inversion method is utilized, high-precision measured data of FVC is necessary for calibration and verification. The measured data mainly depends on the field survey. Although traditional survey can obtain high-precision FVC at the quadrat scale, it consumes a lot of manpower and material resources. Therefore, this causes two problems with the current FVC remote sensing inversion method [51, 89]. On one hand, most FVC inversion studies have little or no measured data [8, 57]. On the other hand, the obtained FVC measured data at the quadrat scale by traditional field survey methods does not match the spatial scale of the satellite remote sensing image pixels ([10, 20, 75, 90]. Consequently, there is urgency to find an efficient field survey method that is both available at a large scale and matches satellite remote sensing image pixels at the spatial scale [12, 56]. In recent years, the gradual maturity of unmanned aerial vehicle (UAV) technology has brought new opportunities. Due to the lower flying height of a UAV, it is not disturbed by atmospheric factors and can take ultra-high-resolution aerial images. In addition, a UAV is portable and inexpensive. It is suitable for FVC field survey under harsh ecological environments [52, 68, 16, 81]. It has been proven in a previous study that UAV technology can not only solve the problem of the mismatch between the FVC measured data and the pixel scale of satellite remote sensing images, but can also access massive high-resolution data with high efficiency [13].

The objective of this study was to find a high-precision and high-efficiency FVC survey and inversion method for analyzing alpine grassland FVC to use in future studies with a focus on: (1) calibrating and evaluating different FVC inversion methods (regression model methods, the pixel dichotomy model, and machine learning algorithms) based on mass FVC measurement data obtained by UAV; (2) constructing a multi-dimensional feature set including original spectral bands, various vegetation indices and topographic factors, and then analyzing the influence of different features on the FVC inversion accuracy through three different feature selection algorithms; (3) tuning parameters for the four machine learning algorithms based on the grid search method to construct an optimized regression model; and (4) quantitatively analyzing the inversion accuracy, computational efficiency, and sensitivity for the sample size of the four machine learning algorithms (MLR, BPNNs, RF, and SVR).

## Study area and data source

### Study area

**Fig. 1:**
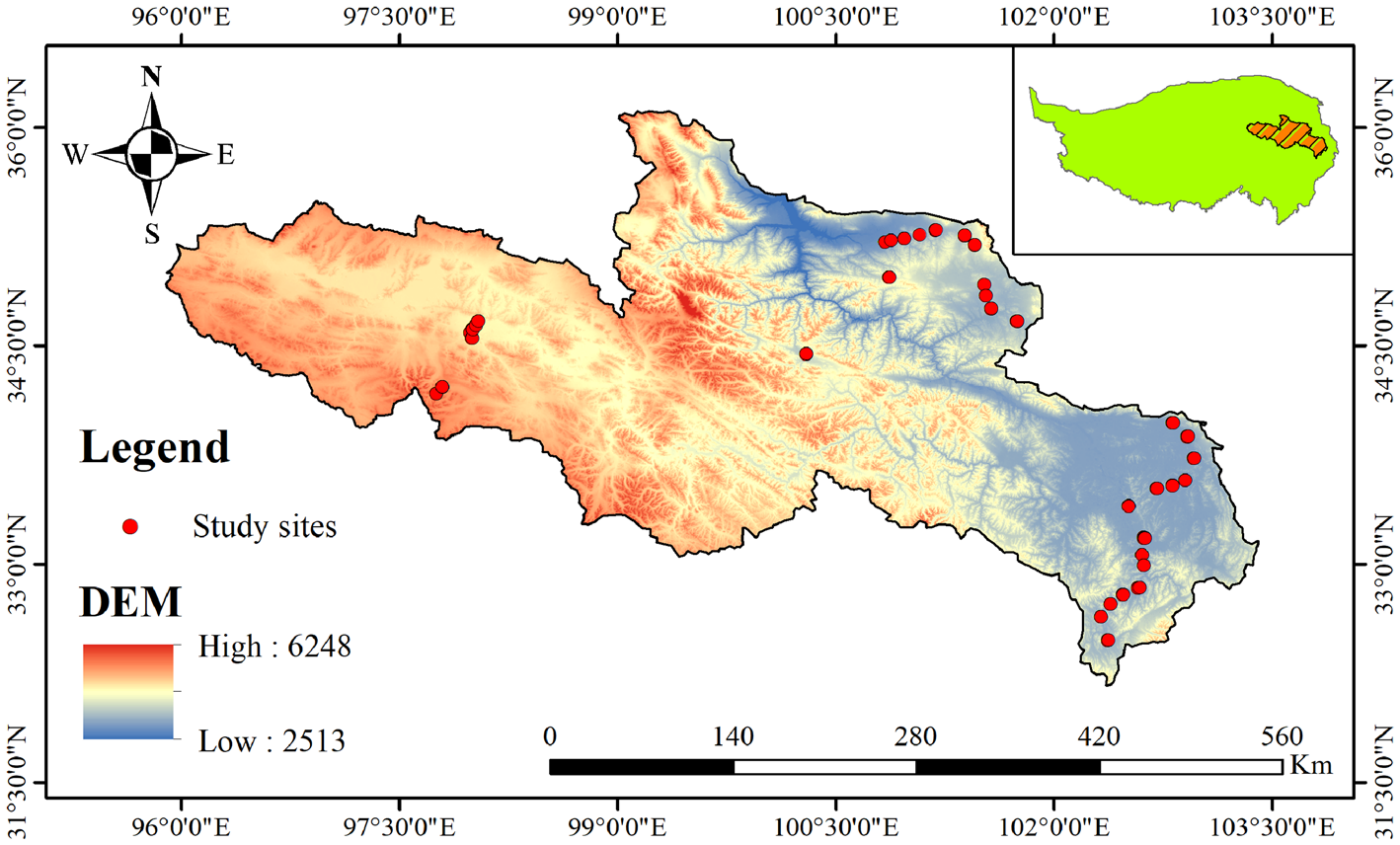
Study area and distribution of observation sites

The source area of the Yellow River Basin (SYRB) is located in the northeastern part of the QTP and is the birthplace of the Yellow River, China's most important freshwater resource. It spans six states and 18 counties in Qinghai, Sichuan, and Gansu provinces, and its total area is approximate 132,000 km^2^ (Fig. 1). Since the average altitude is greater than 4000 m, this area has the environmental characteristics of a low annual average temperature, a large daily temperature difference, long sunshine time, strong solar radiation, and obvious seasonal precipitation. The SYRB is sensitive to climate change, and the ecological environment is fragile. The vegetation types in the SYRB are mainly alpine meadow and alpine steppe, the latter accounting for about 80% of the total land area, which is a microcosm of the QTP. Therefore, high-precision FVC inversion analysis of alpine grassland in the SYRB is vital for local ecological protection and benefits the entire QTP.

### Data source and data preprocessing

#### Field data based on UAV imagery

**Fig. 2:**
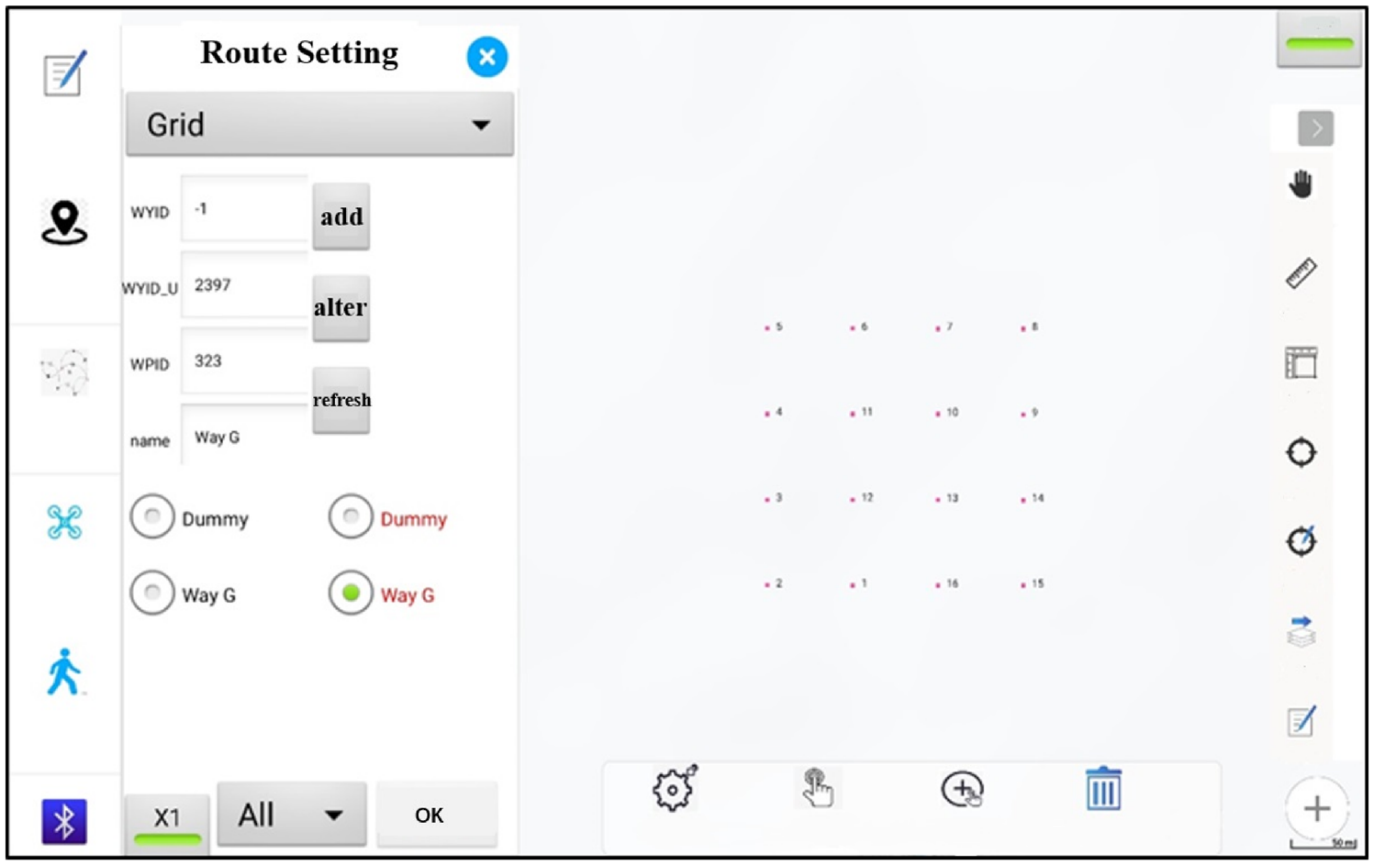
Sampling strategy based on drone aerial photography [50]

In this study, 91 observation sites were set in the SYRB (Fig. 1), and field aerial surveys were carried out from July to August 2015. The 91 observation sites contained different grassland types as well as different underlying surfaces and environmental conditions, and thus they were representative. Our UAV aerial photography operation system, Fragmentation Monitoring and Analysis with aerial Photography (FragMAP) [81] was employed in each observation site to set the UAV flight route. Each observation site contains a route covering the entire monitoring plot and 16 aerial points (Fig. 2). According to the preset parameters to start autonomous flight and aerial photography at a height of 20 m according to the flight route. The spatial resolution of the aerial images was about 1 cm, and the coverage of each aerial image was approximately 30 m × 30 m, which matched the pixel coverage of the Landsat 8 satellite image. The ground truth data is the FVC obtained from each aerial images. The Phantom 3 Professional was used for aerial photography which is a vertical takeoff and landing drone manufactured by SZ DJI Technology Co., Ltd. (Shenzhen, China) that can accurately carry out flight and hovering functions. The GPS/GLONASS dual satellite positioning module was used. The horizontal and vertical accuracy are approximately 1.5 and 0.5 m respectively under hovering, and the gimbal control accuracy is 0.03°. The onboard camera of the UAV was used for photography, which has 12 million camera pixels that can generate a central projection containing three spectral bands of red, green, and blue (RGB); the images were then saved in joint photographic experts group (JPEG) format. During testing, the UAV was flown higher than 4000 m above sea level in the STRB, and the drone could hover for up to 20 min with its maximum flying height exceeding 300 m.

**Fig. 3:**
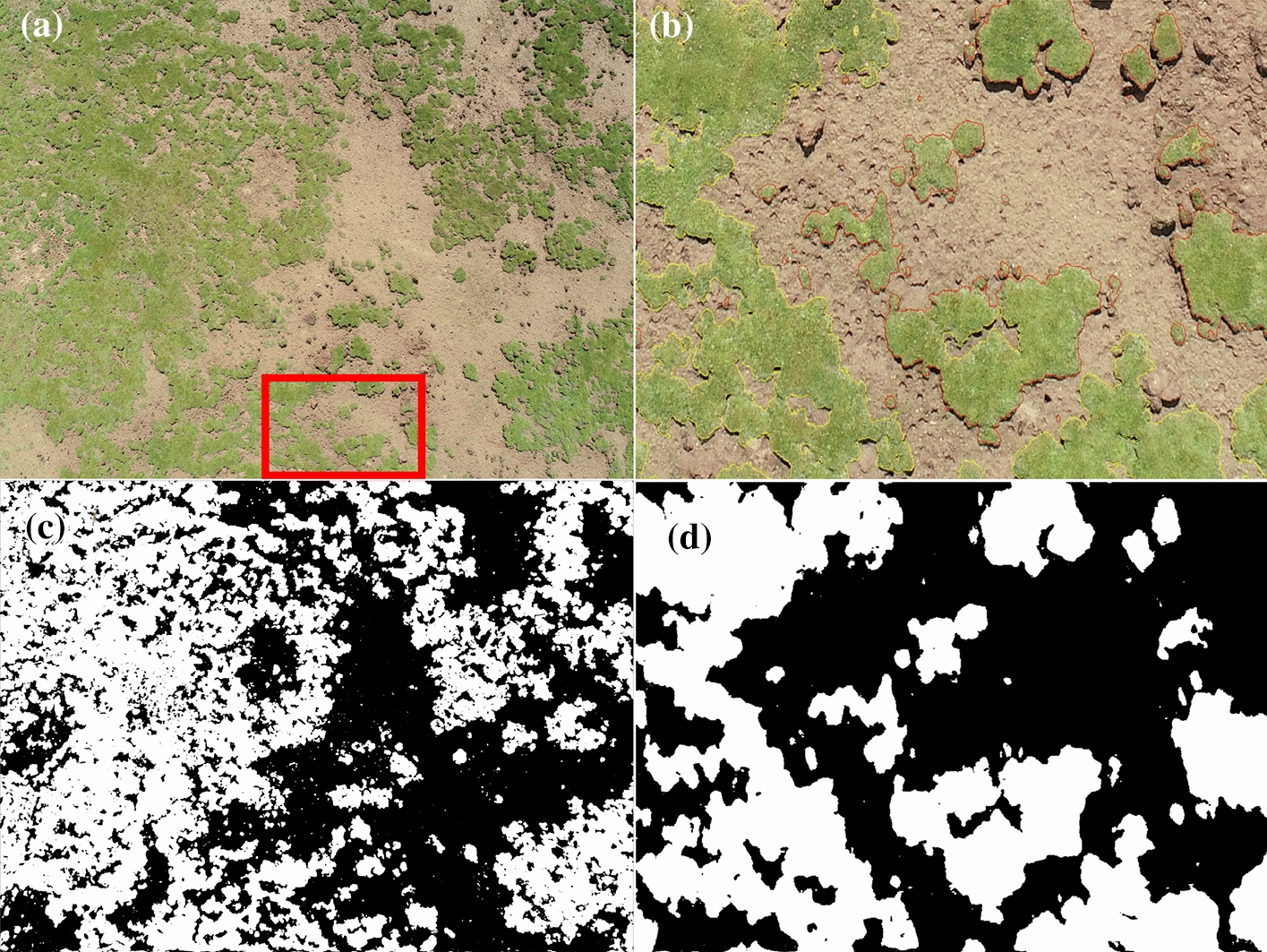
Aerial image and processing effect diagram: **a** aerial image from UAV; **b** EGI segmentation result of aerial image in the red box in (**a**); **c** processing result of aerial image (**a**); **d** processing result of aerial image (**b**)

Previous studies have shown that the threshold segmentation method based on the Excess Green Index (EGI = 2G-R-B, where G, R, and B respectively represent the gray values of the green, red, and blue bands in the image) had good accuracy during the FVC extraction of aerial images [11, 12]. Therefore, the EGI threshold segmentation method was also used in this study to extract FVC information from aerial images. The extraction process of FVC from aerial images was as follows. First, the EGI of each pixel of aerial image was calculated, and an initial value (ranging from 40 to 160 based on our experience) of the EGI threshold was set. And the EGI threshold is determined based on the Java-based FVC Estimator software [12], it is not fixed. Second, if the EGI value of a pixel was greater than the threshold, it was classified as a vegetation pixel, otherwise it was classified as a non-vegetation pixel. Third, the result of the segmentation was superimposed with the original image and judged according to whether the segmentation result was accurate by visual interpretation or not. If the segmentation result was not accurate, the initial threshold value was adjusted until the segmentation result was accurate. Finally, the percentage of vegetation pixels out of the total number of pixels was calculated and determined as the FVC of the image [11, 12] (Fig. 3).

#### Remote sensing data

Landsat 8 Operational Land Imager (Landsat 8 OLI) images were downloaded from the United States Geological Survey (USGS) Earth Explorer website (https://earthexplorer.usgs.gov/). In order to ensure that the acquisition time of the images were consistent with the field investigation time, the images with the cloud cover less than 5% were selected from July 1 to August 31, 2015. A total of 20 Landsat 8 images were needed to cover the entire SYRB. Orthorectification of Landsat 8 images were conducted using the rational polynomial coefficient (RPC) Orthorectification Using Reference Image tool in ENVI 5.3 (Exelis Visual Information Solutions, Boulder, CO, USA) based on the 12.5 m Advanced Land Observing Satellite (ALOS) DEM with an error of less than 0.5 pixels. The Radiometric Correction tool in ENVI 5.3 (Exelis Visual Information Solutions, Boulder, CO, USA) was used for radiation calibration, and the original digital number (DN) value of the Landsat 8 images were converted into spectral reflectance values. Atmospheric correction was performed based on the Fast Line-of-sight Atmospheric Analysis of Spectral Hypercubes (FLAASH) algorithm. Furthermore, the sensor reflectance value was converted into the surface reflectance value. The detailed information of the Landsat 8 OLI images used in this study is shown in Table 1. DEM data generated from the Shuttle Radar Topography Mission (SRTM) at 30 m spatial resolution were download from the USGS, and the slope and aspect were calculated from the DEM in ArcGIS 10.2 (Environmental Systems Research Institute, Redlands, CA, USA).

**Table 1:** Characteristics of Landsat 8 OLI image

Sensor	Bands	Spectral range (nm)	Spatial resolution (m)
Landsat 8 OLI	Coastal (b1)	433–453	30
	Green (b2)	450–515	30
	Blue (b3)	525–600	30
	Red (b4)	630–680	30
	NIR (b5)	845–855	30
	SWIR 1 (b6)	1560–1660	30
	SWIR 2 (b7)	2100–2300	30

## Method

### Regression model method

The regression model method is also called the empirical model method, which is used to establish the relationship between the single band of remote sensing images or the vegetation index obtained by the band calculation and the measured data of the FVC, and then extend the relationship to the study area and finally obtain the FVC of the whole study area [37]. Previous studies have shown that the normalized difference vegetation index (NDVI), enhanced vegetation index (EVI), soil-adjusted vegetation index (SAVI), and modified soil-adjusted vegetation index (MSAVI) have a high correlation with FVC and are often used as driving data in FVC inversion studies [12, 39, 41]. Therefore, we selected these four typical vegetation indices for linear fitting and polynomial fitting (Table 2). The fitting formulas are as follows:

1 $${\text{FVC = }}a \times {\text{VI + }}b$$

2 $${\text{FVC = }}c \times {\text{VI}}^{{2}} { + }d \times {\text{VI + }}e$$

**Table 2:** Multi-dimensional features used in this study

Overview	Vegetation index	References	Calculation formula
Typical vegetation indices	NDVI	[64]	${\text{NDVI = (NIR - R)/(NIR + R)}}$
	EVI	[35]	${\text{EVI = 2}}{.5} \times {\text{(NIR - R)/(NIR + 6}} \times {\text{R - 7}}{.5} \times {\text{B + 1)}}$
	SAVI	[36]	${\text{SAVI = (1 + L}}_{{1}} {)} \times {\text{(NIR - R)/(NIR + R + L}}_{{1}} {)}$
	MSAVI	[59]	${\text{MSAVI = }}\left[ {{2} \times {\text{NIR + 1 - }}\sqrt {{(2} \times {\text{NIR + 1)}}^{{2}} { - 8} \times {\text{(NIR - R)}}} } \right]{/2}$
Added vegetation indices	Simple Ratio (SR)	[5]	${\text{SR = NIR/R}}$
	Atmospherically Resistant Vegetation Index (ARVI)	[42]	${\text{ARVI = }}\left[ {{\text{NIR - }}\left( {{2} \times {\text{R - B}}} \right)} \right]{/}\left[ {{\text{NIR + }}\left( {{2} \times {\text{R - B}}} \right)} \right]$
	Difference Vegetation Index (DVI)	[71]	${\text{DVI = NIR - R}}$
	Global Environmental Monitoring index (GEMI)	[58]	$\left\{ {_{{{\text{eta = }}\left[ {{\text{2(NIR}}^{{2}} {\text{ - R}}^{{2}} {) + 1}{\text{.5}} \times {\text{NIR + 0}}{.5} \times {\text{R}}} \right]{\text{/(NIR + R + 0}}{.5)}}}^{{{\text{GEMI = eta(1 - 0}}{.25} \times {\text{eta) - (R - 0}}{\text{.125)/(1 - R)}}}} } \right.$
	Green Atmospherically Resistant Index (GARI)	[29]	${\text{GARI = }}\left\{ {{\text{NIR - }}\left[ {{1}{\text{.7}} \times {\text{(B - R)}}} \right]} \right\}{/}\left\{ {{\text{NIR + }}\left[ {{1}{\text{.7}} \times {\text{(B - R)}}} \right]} \right\}$
	Green Difference Vegetation Index (GDVI)	[67]	${\text{GDVI = NIR - G}}$
	Green Normalized Difference Vegetation Index (GNDVI)	[30]	${\text{GNDVI = (NIR - G)/(NIR + G)}}$
	Green Ratio Vegetation Index (GRVI)	[67]	${\text{GRVI = NIR/G}}$
	Green Vegetation Index (GVI)	[43]	$$\left\{ \begin{gathered} {\text{GVI = ( - 0}}{.2848} \times {\text{B) + ( - 0}}{.2435} \times {\text{B) + ( - 0}}{.5436} \times {\text{R) + }} \hfill \\ { (0}{\text{.7243}} \times {\text{NIR) + (0}}{.0840} \times {\text{SWIR1) + }} \hfill \\ { ( - 0}{\text{.1800}} \times {\text{SWIR2)}} \hfill \\ \end{gathered} \right.$$
	Infrared Percentage Vegetation Index (IPVI)	[19]	${\text{IPVI = NIR/(NIR + R)}}$
	Leaf Area Index (LAI)	[6]	${\text{LAI = 3}}{.618} \times {\text{EVI - 0}}{.118}$
	Modified Non-Linear Index (MNLI)	[80]	${\text{MNLI = (NIR}}^{{2}} {\text{ - R)}} \times {\text{(1 + L}}_{{2}} {\text{)/(NIR}}^{{2}} {\text{ + R + L}}_{{2}} {)}$
	Modified Simple Ratio (MSR)	[15]	${\text{MSR = (NIR/R - 1)/(}}\sqrt {\text{NIR/R}} { + 1)}$
	Non-Linear Index (NLI)	[31]	${\text{NLI = (NIR}}^{{2}} {\text{ - R)/(NIR}}^{{2}} {\text{ + R)}}$
	Optimized Soil Adjusted Vegetation Index (OSAVI)	[62]	${\text{OSAVI = }}\left[ {{1}{\text{.5}} \times {\text{(NIR - R)}}} \right]{\text{/(NIR + R + 0}}{.16)}$
	Renormalized Difference Vegetation Index (RDVI)	[63]	${\text{RDVI = (NIR - R)/}}\sqrt {\text{NIR + R}}$
	Transformed Difference Vegetation Index (TDVI)	[3]	${\text{TDVI = }}\sqrt {{0}{\text{.5 + (NIR - R)/(NIR + R)}}}$
	Visible Atmospherically Resistant Index (VARI)	[28]	${\text{VARI = (G - R)/(G + R - B)}}$
Topographical factors	DEM	–	SRTM DEM
	slope		
	aspect		

^1^L_1_, and L_2_ are soil adjustment factor, which are considered by [59, 80] to be the best value of 0.5. NIR represent the reflectance of near-infrared band

where FVC is fractional vegetation cover; VI is vegetation index; *a* is the slope of linear fitting; *b* and *e* are the intercepts of linear fitting and polynomial fitting, respectively; and *c* and *d* are the parameter estimation values of polynomial fitting.

### Pixel dichotomy model

The pixel dichotomy model is currently the most widely used method for estimating FVC. It assumes that the pixel information received by the satellite sensor is composed of vegetation and soil, and FVC is the percentage of a pixel occupied by vegetation. The NDVI is considered to be a good indicator for FVC, so the pixel dichotomy model with the NDVI as the input parameter was used in this study to estimate the FVC of the SYRB [22, 70]. The formula is as follows:

3 $${\text{FVC = }}\frac{{{\text{NDVI - NDVI}}_{{\text{S}}} }}{{{\text{NDVI}}_{{\text{V}}} {\text{ - NDVI}}_{{\text{S}}} }}$$

where NDVI_s_ and NDVI_v_ are NDVI values in the area that were completely covered by soil and vegetation, respectively.

In this study, a total of two sets of NDVI_s_ and NDVI_v_ values were used to estimate the FVC of the SYRB based on the pixel dichotomy model: (1) the values of pure vegetation pixels and pure soil pixels based on the statistical results of the ecological function area in the existing literature [40] (NDVI_v_ = 0.837, NDVI_s_ = 0.164) and (2) the values of pure vegetation pixels and pure soil pixels determined by 95% confidence intervals [9] (NDVI_v_ = 0.882, NDVI_s_ = 0.067).

### Machine learning algorithms

For all machine learning algorithms, the input layers are the multi-dimensional features, and the output layer is the FVC results. Firstly, we normalized all the features in the multi-dimensional feature set (original spectral bands, multiple vegetation indices, and topographical factors) whose values were not in the range of 0–1 in R before implementing the machine learning algorithm. And we performed a random cut of the dataset, with 70% of the dataset used for training and 30% for validation. Then, the machine learning algorithm was trained through the training dataset to build the internal relationship between the multi-dimensional features and FVC measured data. Finally, the FVC inversion accuracy was evaluated, and the accuracy was verified through the test training set based on the trained machine learning algorithm.

#### Optimized MLR

MLR is based on two or more variables for regression analysis. It is considered to be an effective and more realistic statistical analysis method, which is widely used in the field of vegetation physiological structure parameter inversion [32]. The MLR model in this study was constructed and optimized based on the "stats" package of the R language platform and the multiple linear formula is as follows:

$${\text{FVC}} = a + b_{1} {\text{x}}_{1} + b_{2} {\text{x}}_{2} + \cdots + b_{n} {\text{x}}_{n}$$

where a, b_1_, b_2_, … b_n_ are parameters to be optimized, FVC is the result of fractional vegetation cover predicted by MLR, and x_1_, x_2_ … x_n_ are feature variables in the multi-dimensional feature set.

#### Optimized BPNNs

BPNNs are a concept proposed by Rumelhart et al. [65], which is a multi-layer feed forward neural network trained according to the backward propagation algorithm of error. BPNNs are one of the machine learning algorithms widely used in the inversion of physiological structure parameters of vegetation [47]. BPNNs in this study were based on the "neuralnet" package of the R language platform. The weight attenuation parameter and threshold value in the BPNN algorithm were set to 0.01. In addition, the grid search method was used to tune the number of hidden layers of the BPNN algorithm and the number of neurons in each hidden layer. The setting range of the number of hidden layers was 1–5, and the setting range of the number of neurons in each hidden layer was 1- 10. After cross-validating 10 times, the model training results showed that the optimal number of hidden layers was two, the optimal number of neurons in the first hidden layer was two, and the optimal number of neurons in the second hidden layer was four. The hidden layer activation function was set to tansig after the optimization of sigmoid, the output layer transfer function was set to purelin to make the constructed BPNNs suitable for the linear model, and trainlm was selected as the training function.

#### Optimized SVR

Support vector machines (SVMs) are new machine learning algorithms based on the statistical theory that one is able to achieve high accuracy when solving the classification and regression problems of high-dimensional features without needing to rely on all the data to make hyperplane decisions [18]. Support vector regression (SVR) is the performance of the SVM method for regression. [84]. SVR in this study is based on the LIBSVM interface in the "e1071" package of the R language platform, and the FVC for the source area in the Yellow River Basin was predicted via regression. The SVM type was set to e-SVR, the loss function P was 0.01, and the kernel function type was radial basis function (RBF). In order to achieve a better prediction result for SVR, the grid search method was used to optimize the RBF kernel parameter (gamma) and penalty coefficient (cost) in the SVR algorithm. The setting range of gamma was set to 0.5–4, the setting range of cost was set to 0.5–8, and the step length of gamma and cost was 0.5. After cross-validating 10 times, the model training results showed that the optimal gamma and cost values were 0.5 and four, respectively.

#### Optimized RF

The RF algorithm was proposed by Breiman in 2001. This algorithm is based on the bagging integrated learning method, which integrates multiple decision trees into a forest and combines them to predict the final result [7]. The RF algorithm has a good anti-noise ability. It is simple, fast, easy to achieve parallelization, and avoids overfitting to a certain extent [49]. In the RF regression algorithm, a decision tree represents a set of constraints. These conditions are organized hierarchically and applied from the root to the leaves in succession. Two parameters of the RF algorithm need to be defined: the number of decision trees (ntree) and the number of characteristic variables required to create branches (mtry). Based on the “randomForest” package of the R language platform, the grid search method was used to optimize the parameters of mtry and ntree in the RF regression algorithm. The setting range of mtry was set to 1–31 with a step size of 1, and the setting range of ntree was set to 100–2,000 with a step size of 100. After cross-validating 10 times, the model training results showed that the optimal mtry and ntree values were 13 and 1200, respectively.

### Feature selection

Feature selection directly affects the training speed and prediction performance of machine learning algorithms, which enables us to have a better understanding of the true distribution behind the multi-dimensional feature set. It is an important means to eliminate redundant information. If a feature is considered by different variable selection algorithms to have an important effect on the accuracy of the inversion result, it is a feature worthy of attention. In this study, we used Boruta, Sequential Forward Selection (SFS), and Permutation Importance-Recursive Feature Elimination (PI-RFE), which are three different feature selection methods applied to multi-dimensional feature sets to determine the appropriate dimension, eliminate redundant features, and obtain satisfactory FVC inversion accuracy.

Boruta is a fully correlated feature selection algorithm, and its main objective is to select all feature sets related to the dependent variable [45]. The SFS algorithm is a kind of greedy search algorithm that is used to reduce the initial multi-dimensional feature set to a low-dimensional feature set [21]. The main idea of the SFS algorithm is to automatically select the subset of features most relevant to the dependent variable, and improve calculation efficiency and reduce generalization errors by removing irrelevant features. PI-RFE is an optimized RFE algorithm constructed in this research. RFE is a greedy algorithm that finds the best feature subset [27]. The main idea of the RFE algorithm is to repeatedly build the model to select the best feature, and then repeat this process in the remaining features until all the features are evaluted. PI sets a feature in the multi-dimensional dataset as unavailable, and characterizes the importance of the feature through the decrease in accuracy of the inversion model [2]. In this study, the built-in weight parameters of the RFE algorithm were replaced with the important variables determined by PI.

### Accuracy assessment

In this study, the data set was randomly divided. Seventy percent was used as model training data while the remaining 30% was used as model test data. The correlation between the inversion results of the model test data and the measured results of FVC was analyzed. The determination coefficient (R^2^) and the root mean square error (RMSE) were considered to be reasonable evaluation indicators of accuracy. The performance of the above-mentioned inversion models of FVC was evaluated by the values of R^2^ and RMSE. They were calculated by Eqs. (5) and (6) below:

5 $${\text{R}}^{{2}} = 1 - \frac{{\sum\limits_{i = 1}^{n} {(S_{i} - S_{i}^{^{\prime}} )^{2} } }}{{\sum\limits_{i = 1}^{n} {(S_{i} - \mathop {S_{i} }\limits^{\_} )^{2} } }}$$

6 $${\text{RMSE = }}\sqrt {\frac{1}{n}\mathop \sum \limits_{i = 1}^{n} (S_{i} - S_{i}^{^{\prime}} )^{2} }$$

where n represents the number of samples, *S*_*i*_ represents the measured values of sites, *S*_*i*_*’* represents the predicted values of the model, and $\overline{{\text{S} }_{\text{i}}}$ represents the mean of the predicted values of the model. Generally, the higher the value of R^2^, the smaller the value of the RMSE, indicating that the model performance was better.

In order to evaluate the sensitivity to the training sample size of the four machine learning algorithms, the R^2^ and RMSE between the training samples and the verification samples were obtained. The sample data set was randomly selected from the total training samples (270), and the training sample for the minimum data set was 30. This was sequentially incremented by 30 until the training sample was 270, with a total of nine data sets.

## Results

### Regression model method

Linear fitting showed that there was a good relationship between the four vegetation indices (NDVI, EVI, SAVI, and MSAVI) and the measured FVC (Table 3). The FVC obtained by linear fitting inversion showed that the NDVI fitting had the highest accuracy (R^2^: 0.717, RMSE: 10.8%), followed by SAVI (R^2^: 0.665, RMSE: 11.4%), MSAVI (R^2^: 0.642, RMSE: 11.7%), and EVI (R^2^: 0.635, RMSE: 12.1%), as shown in Table 4. The polynomial fitting relationship between vegetation indices and measured FVC was better than the linear fitting (Table 3). The FVC obtained by polynomial fitting inversion showed that the NDVI fitting had the highest accuracy (R^2^: 0.745, RMSE: 9.8%), followed by SAVI (R^2^: 0.725, RMSE: 10.3%), MSAVI (R^2^: 0.724, RMSE: 10.5%), and EVI (R^2^: 0.715, RMSE: 11.8%), as shown in Table 4.

**Table 3:** Linear and polynomial fitting relationships and inversion accuracy between the four vegetation indices (VIs) and FVC

VIs	Linear fitting			Polynomial fitting		
	**Fitting formula**	**R**^**2**^	**RMSE (%)**	**Fitting formula**	**R**^**2**^	**RMSE (%)**
NDVI	y = 1.276x-0.008	0.717	10.8	y = -2.810x^2^ + 4.536x-0.859	0.745	9.8
EVI	y = 1.212x + 0.269	0.635	12.1	y = -4.955x^2^ + 5.673x-0.636	0.715	11.8
SAVI	y = 1.482x + 0.172	0.665	11.4	y = -6.775x^2^ + 7.180x-0.921	0.725	10.3
MSAVI	y = 1.210x + 0.299	0.642	11.7	y = -5.437x^2^ + 5.881x-0.600	0.724	10.5

**Table 4:** Features selected by different feature selection algorithms

Algorithm name	Selected features
Boruta	DEM, VARI, slope, ARVI, NDVI, SR, TDVI, IPVI, GARI, b7, MSR, OSAVI, b2, b4, NLI, MNLI, GNDVI, GRVI, RDVI, b6, aspect, EVI
SFS	DEM, slope, VARI, b7, ARVI, b4, b2, OSAVI, GARI, aspect, b6, IPVI, SR, MSR, NDVI
PI-RFE	DEM, VARI, slope, b7, aspect, b2, b4, ARVI, OSAVI, NDVI, SR, GARI, TDVI, NDVI, IPVI, b1, MSR, b6

### Pixel dichotomy model

**Fig. 4:**
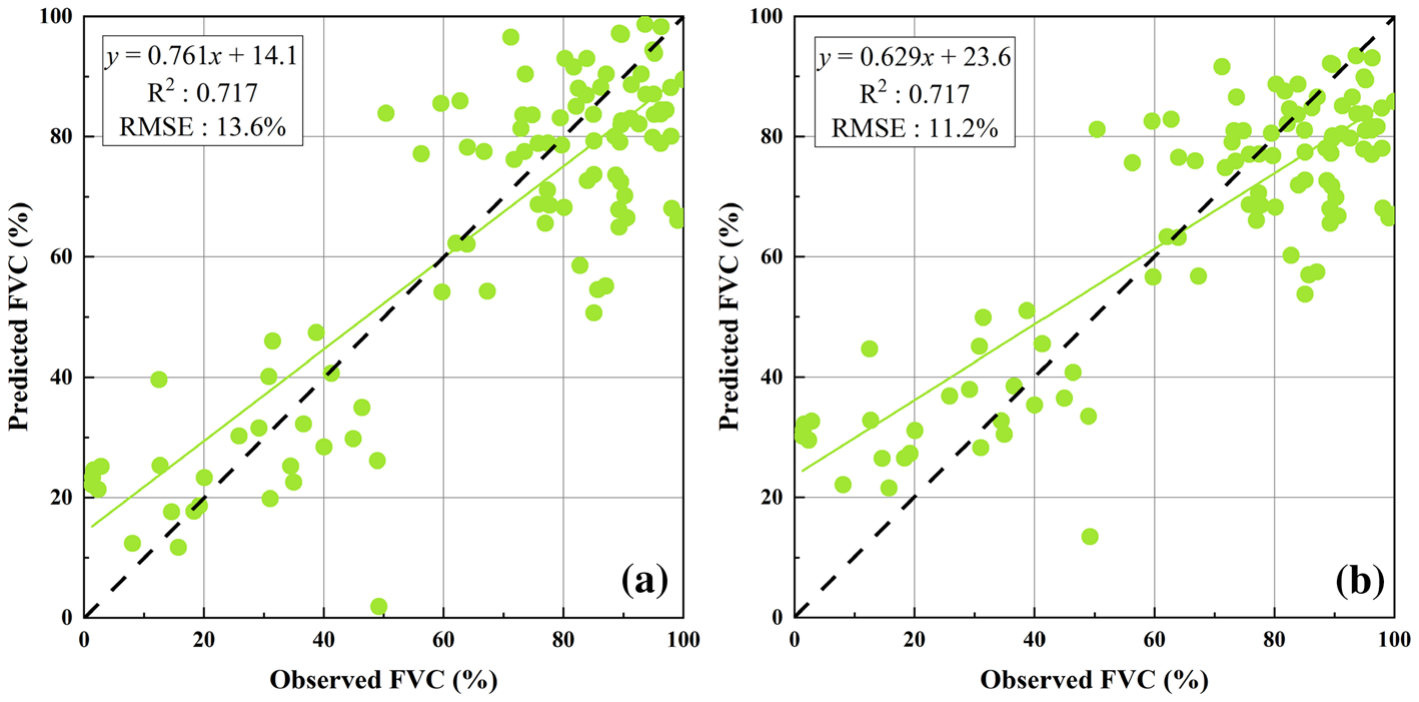
The accuracy of inversion of FVC **a** based on ecological function area **b** based on 95% confidence interval

The FVC inversion results based on the pixel dichotomy model had good inversion accuracy. The R^2^ based on the ecological function area and based on a 95% confidence interval were both 0.717, while the RMSE of latter was lower than that of the former (Fig. 4).

### Machine learning algorithms

#### FVC evaluation using four typical vegetation indices

**Fig. 5:**
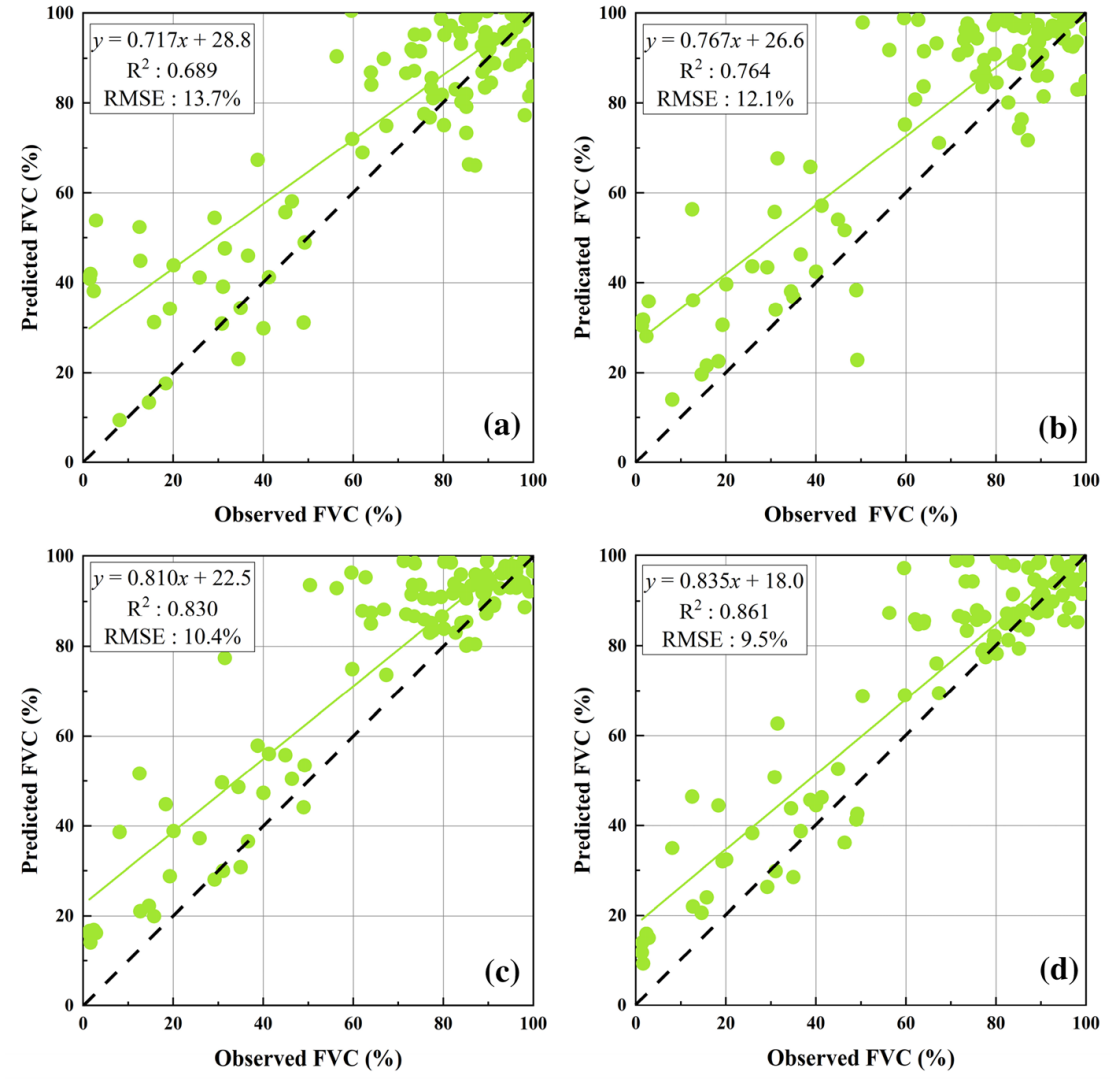
Evaluation accuracy when the driving data were four commonly used vegetation indices and spectral reflectance bands **a** MLR; **b** BPNNs; **c** SVR; **d** RF

FVC inversion results of the four machine learning algorithms showed that when the driving data were the four commonly used vegetation indices, the FVC inversion accuracy was higher than in the regression model method and pixel dichotomy model. The RF regression algorithm (R^2^: 0.861, RMSE: 9.5%) and SVR (R^2^: 0.830, RMSE: 10.4%) showed the highest accuracy, followed by BPNNs (R^2^: 0.764, RMSE: 12.1%) and MLR (R^2^: 0.689, RMSE: 13.7%), as shown in Fig. 5.

#### FVC estimation using a multi-dimensional feature set

**Fig. 6:**
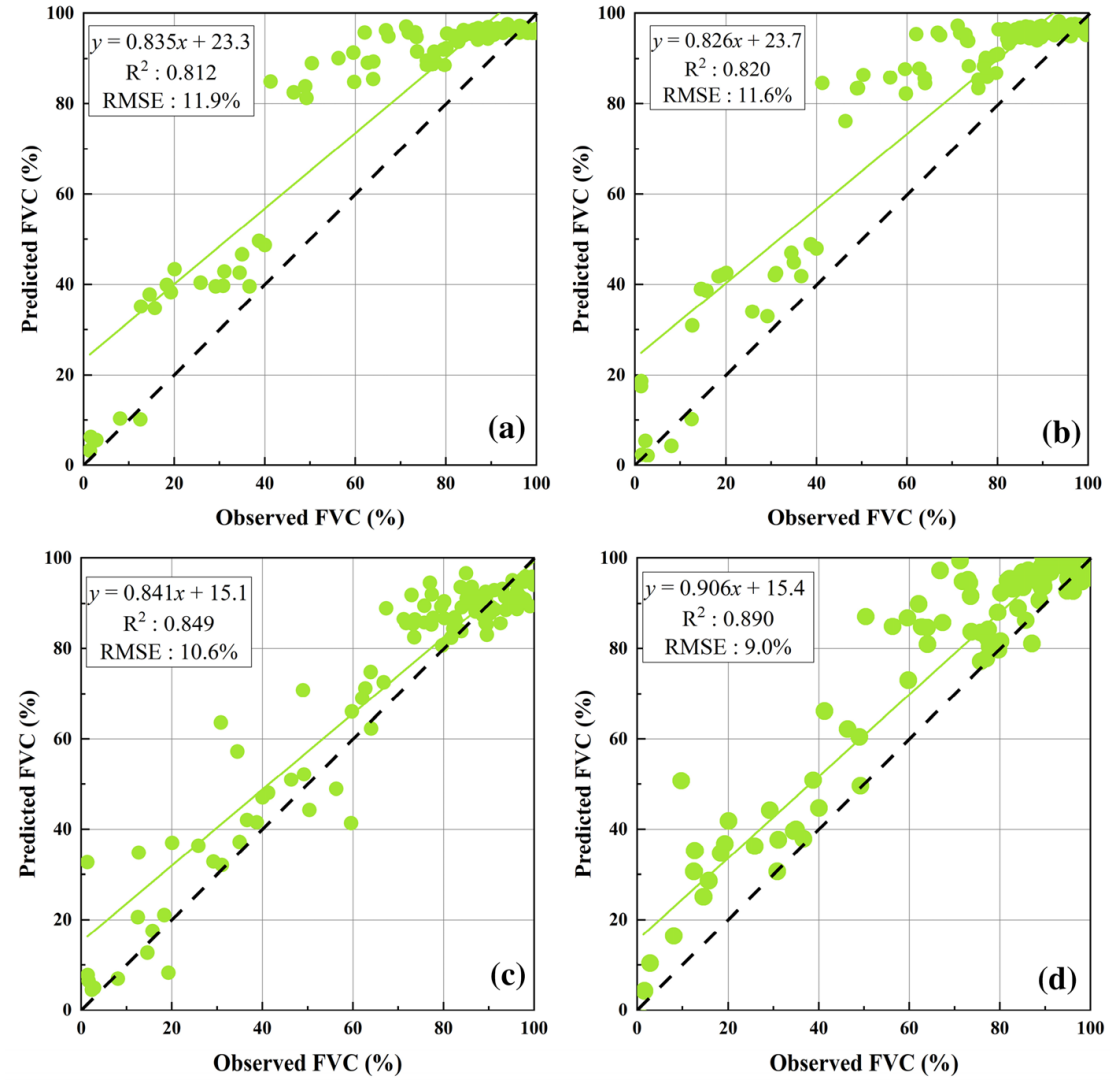
Evaluation accuracy when the driving data are a multi-dimensional feature set **a** MLR; **b** BPNNs; **c** SVR; **d** RF

FVC inversion results showed that the accuracy of the four machine learning algorithms had been improved after adding original spectral bands, 18 vegetation indices and DEM, aspect, and slope (Fig. 6). The R^2^ was greater than 0.81 and the RMSE was less than 11.9%. RF had the highest inversion accuracy among the four machine learning algorithms with an R^2^: 0.890 and RMSE: 9.0%, followed by SVR (R^2^: 0.849, RMSE: 10.6%) and BPNNs (R^2^: 0.820, RMSE: 11.6%). MLR had the lowest inversion accuracy with an R^2^: 0.812 and RMSE: 11.9%.

#### Optimal feature subset and feature importance

**Fig. 7:**
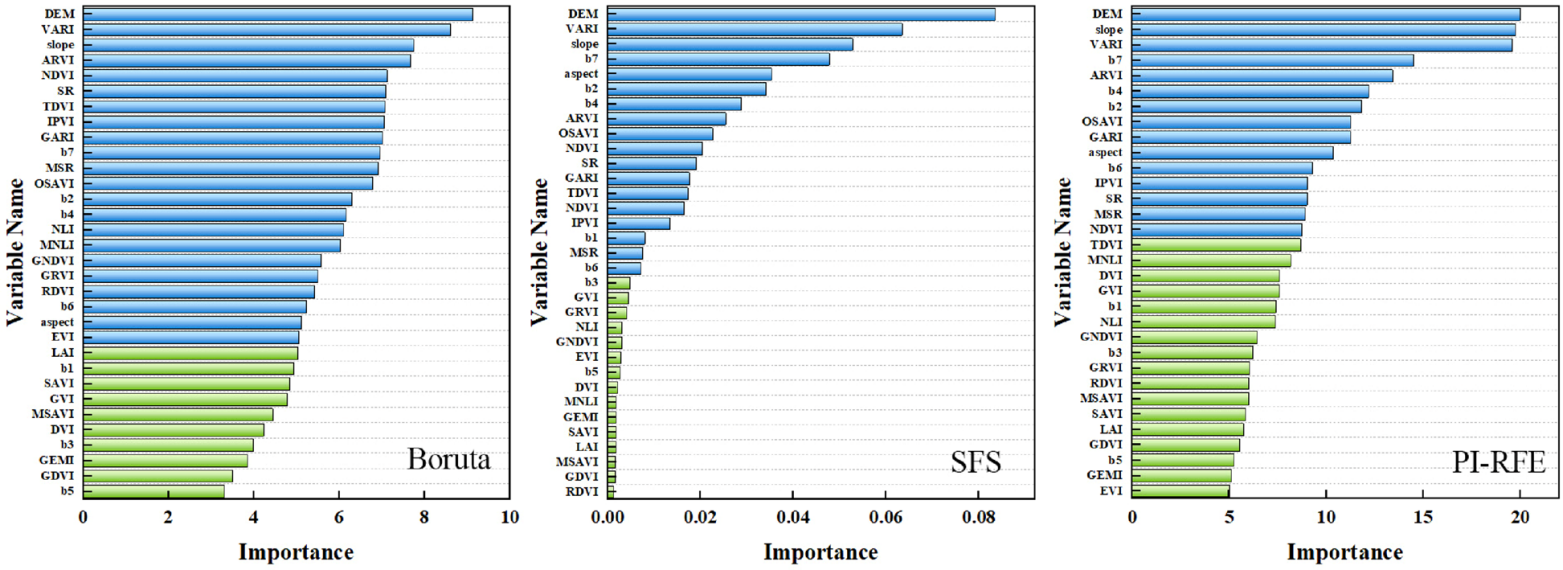
Multi-dimensional feature importance evaluation based on three feature selection algorithms (blue and green represent important and unimportant features, respectively.)

The results of feature selection for multi-dimensional feature sets based on three feature selection algorithms showed that 22 features in the Boruta model were retained: DEM, VARI, slope, ARVI, NDVI, SR, TDVI, IPVI, GARI, b7, MSR, OSAVI, b2, b4, NLI, MNLI, GNDVI, GRVI, RDVI, b6, aspect, and EVI. In the SFS algorithm, 15 features were retained: DEM, slope, VARI, b7, ARVI, b4, b2, OSAVI, GARI, aspect, b6, IPVI, SR, MSR, and NDVI. In the PI-RFE algorithm, 18 features were retained: DEM, VARI, slope, b7, aspect, b2, b4, ARVI, OSAVI, NDVI, SR, GARI, TDVI, NDVI, IPVI, b1, MSR, and b6. Across all feature selection algorithms, it was consistently revealed that the most important feature was DEM, followed by slope and VARI. A comprehensive comparison of the three algorithms found that among vegetation indices, eight vegetation indices (VARI, ARVI, SR, NDVI, IPVI, GARI, MSR, and OSAVI) were selected as important features. Among reflectance bands, four reflectance bands (b2, b4, b6, and b7) were selected as important features. Among topographical factors, DEM, slope and aspect were all retained as important features (Fig. 7).

#### Performance evaluation of feature selection algorithms

**Fig. 8:**
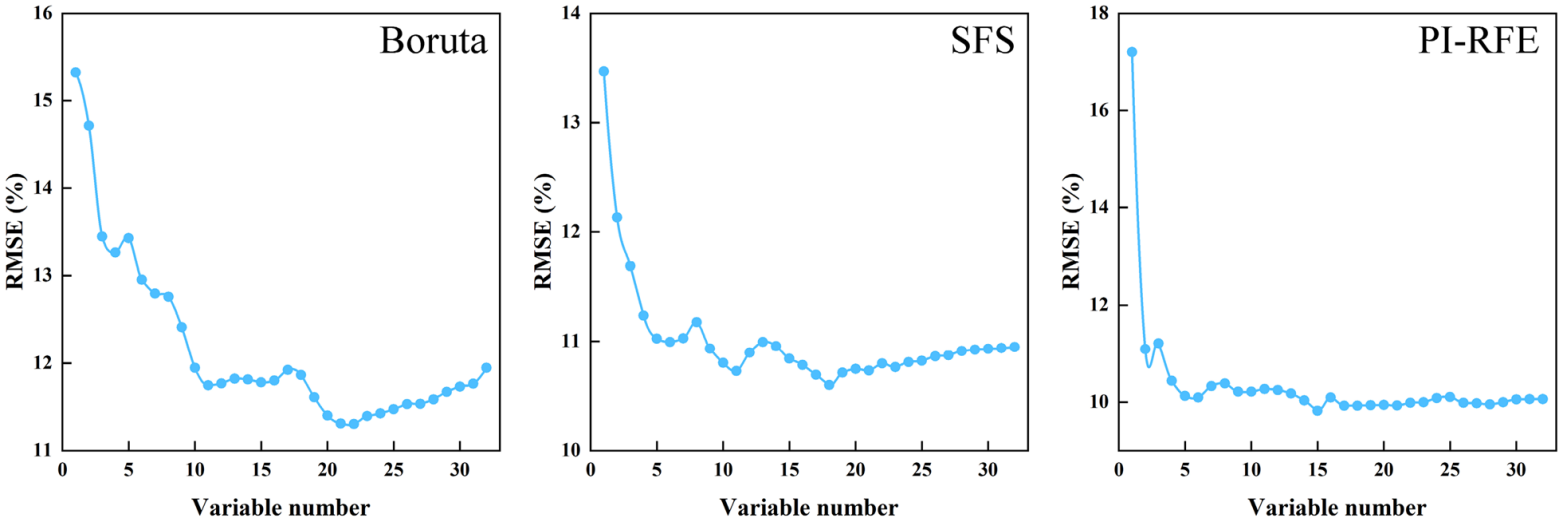
Trend of RMSE with the number of features for three variable selection algorithms

The variation trend of RMSE with the number of features in the three different feature selection algorithms is shown in Fig. 8. While the Boruta-based feature selection was being employed, the RMSE dropped sharply to 11.7% when the number of features was 1 to 11; the fluctuation then decreased, and when the number of features was 22, the RMSE achieved its lowest value of 11.3%. During SFS-based feature selection, the RMSE dropped sharply to 11.0% when the number of features was 1 to 5; the fluctuation then decreased, and when the number of features was 18, the RMSE achieved its lowest value of 10.6%. When performing PI-RFE-based feature selection, the RMSE dropped sharply to 10.1% when the number of features was 1 to 6 and then maintained a steady downward trend; when the number of features was 15, the RMSE achieved its lowest value of 9.8%.

#### FVC inversion based on optimized machine learning algorithms and feature subset

**Fig. 9:**
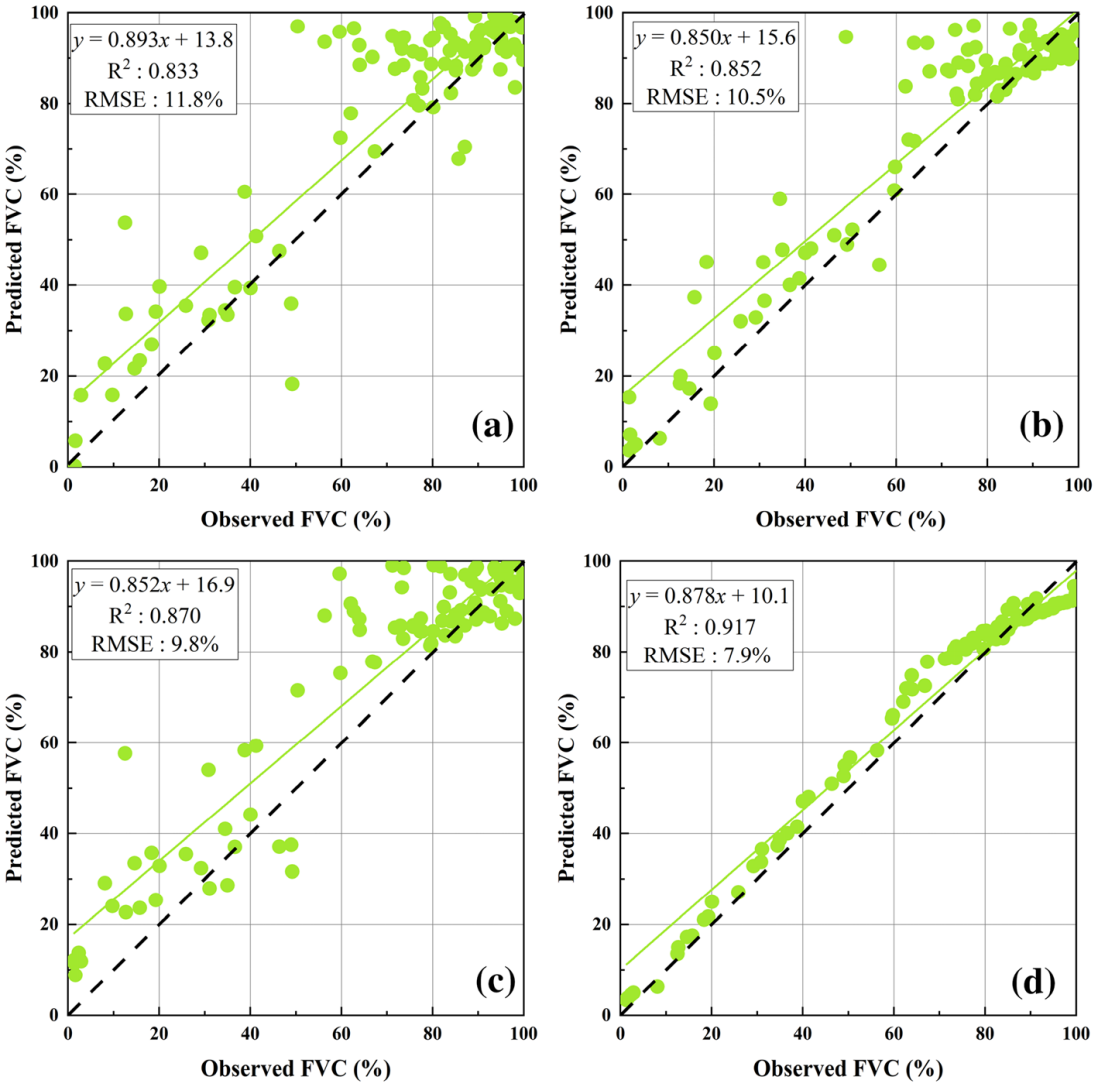
Evaluation accuracy when using optimized machine learning algorithms and optimized feature subset **a** MLR; **b** BPNNs; **c** SVR; **d** RF

The FVC inversion results of the four machine learning algorithms after parameter tuning showed that when the driving data was the optimized feature subset from PI-RFE-based variable selection, the accuracy of FVC inversion was greatly improved compared with the use of multi-dimensional feature sets and original machine learning algorithms. (R^2^ was greater than 0.833, RMSE was less than 11.8%), as shown in Fig. 9. RF had the highest inversion accuracy among the four machine learning algorithms with an R^2^: 0.917 and RMSE: 7.9%, followed by SVR (R^2^: 0.870, RMSE: 9.8%) and BPNNs (R^2^: 0.852, RMSE: 10.5%). MLR had the lowest inversion accuracy with an R^2^: 0.833 and RMSE: 11.8%.

#### Computational efficiency

There were obvious differences between the training and estimation times of the four machine learning algorithms (Table 5). SVR had the longest training and estimation time (168.84 and 182.82 s), followed by MLR and BPNNs. Both the training and estimation times of the RF regression algorithm were the shortest, at 87.51 and 99.35 s, respectively.

**Table 5:** Training/estimation time required for one iteration of the machine learning algorithms

	R^2^	RMSE (%)	Training time	Estimation time
MLR	0.833	11.8	140.58 s	163.76 s
BPNNs	0.852	10.5	128.48 s	143.45 s
SVR	0.870	9.8	168.84 s	182.82 s
RF	0.917	7.9	87.51 s	99.35 s

#### Sensitivity to training sample size

**Fig. 10:**
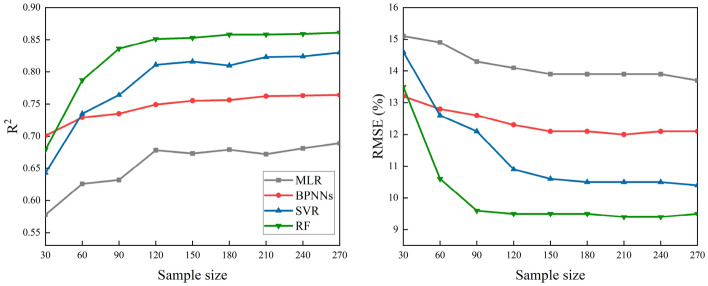
Performance of four machine learning algorithms with different training sample sizes

The sensitivity test of the training sample size of the four machine learning algorithms showed that as the training sample size increased, the sensitivity difference of the algorithms is obvious (Fig. 10). When the training sample size was small, the four machine learning algorithms were more sensitive to changes in training sample size. The RF and SVR regression algorithms were more sensitive to the training sample size than MLR and BPNNs. With an increase in the training sample size, however, their sensitivity gradually decreased. When the training sample was greater than 120, the sensitivity of the four machine learning algorithms tended to stabilize, where R^2^ and the RMSE did not noticeably change with an increase in training sample size.

In addition, when the training sample size was small and fixed, the sensitivities of the four machine learning algorithms to the training sample size were different (Fig. 10). Among them, the RF regression algorithm was the most robust and MLR was the worst. However, when the training sample size was large, the sensitivity difference of the four machine learning algorithms to the training sample size was not obvious.

## Discussion

### Accuracy evaluation of different FVC inversion methods

Previous studies had shown that to some extent, the EVI, SAVI, and MSAVI could explain changes in the optical characteristics of the background and correct the effects of atmospheric and soil backgrounds, which was not found with the NDVI [1]. However, we found that the NDVI achieved the highest FVC inversion accuracy (R^2^: 0.717, RMSE: 11.7%) among the four vegetation indices (NDVI, EVI, SAVI, and MSAVI), which indicated that the NDVI was more suitable for FVC inversion in alpine grassland than the EVI, SAVI, and MSAVI. The specific reason for this may be due to the limited biomass per unit area in the alpine grassland and had no obvious influence on the NDVI saturation phenomenon [14].

The pixel dichotomy model is another commonly used method for FVC remote sensing inversion. The key to the construction of the pixel dichotomy model is the determination of the end-members. Generally, NDVI_s_ and NDVI_v_ determined by measured spectral data, would obtain a higher FVC inversion accuracy. However, the special climate and topography of the QTP led to some deviations in data collection which affected the final inversion results [46]. In addition, the final member determination was easily influenced by factors such as soil type, vegetation type, chlorophyll content, etc. The NDVI_s_ and NDVI_v_ determined by the statistical results of ecological function areas and the 95% confidence interval proved to have high FVC inversion accuracy [4, 40]. This study proved that the pixel dichotomy model based on 95% confidence intervals for NDVI_s_ and NDVI_v_ was more suitable for FVC inversion in alpine grassland than that based on the statistical results of ecological function areas. The reason for this may be that the method of establishing the NDVI_s_ and NDVI_v_ based on the statistical results of the ecological function area was a universal method, and the method of establishing the NDVI_s_ and NDVI_v_ based on the 95% confidence interval was derived from the statistics of the NDVI value in the study area. Therefore, when applied to a certain vegetation type or a certain area, the former FVC inversion accuracy would be lower than the latter.

In recent years, machine learning algorithms have been widely used in the field of vegetation physiological parameter inversion based on satellite remote sensing images [55, 39]. In this study, four commonly used vegetation indices (NDVI, EVI, SAVI, and MSAVI) were used as driving data for FVC inversion, and the results showed that the RF algorithm obtained the highest inversion accuracy (R^2^: 0.861, RMSE: 9.5%) while MLR had the lowest inversion accuracy (R^2^: 0.689, RMSE: 13.7%) during FVC inversion of the four machine learning algorithms. Nonetheless, SVR and BPNNs also had good inversion accuracy. Our findings suggested that the performance of machine learning algorithms in the FVC inversion of alpine grassland was better than the regression model method and the pixel dichotomy model, and these could be used for high-precision FVC inversion of alpine grassland [85].

### Factors that influence FVC inversion in machine learning algorithms

Driving data directly affected the accuracy of the prediction results for machine learning algorithms [69]. This study comprehensively selected original spectral reflectance bands, topographic factors, and multiple vegetation indices as the driving data for FVC inversion. Compared with the four commonly used vegetation indices, the alpine grassland FVC inversion accuracy based on the four machine learning algorithms had been obviously improved. Among them, the FVC inversion accuracy of the MLR algorithm was improved the most, which indicated that topographic factors and various vegetation indices also have a high correlation with the FVC of alpine grassland, while the internal factor of how different features in the driving data affect the FVC inversion results of alpine grassland cannot be explained by the “black box model” of machine learning algorithms [86].

Therefore, we further quantitatively evaluated the importance of features in the driving data based on the index of the influence of feature variables on the accuracy of FVC inversion. The results showed that the four commonly used vegetation indices and original spectral reflectance bands are not the most ideal driving data. In fact, three topographic factors play a more important role in the accuracy of the inversion results than other features. The importance of DEM ranked first among all features, because changes in altitude directly affected temperature, precipitation, solar radiation and other factors closely related to the vegetation growth status. In addition, vegetation indices such as VARI, ARVI, and SR were also more important than the four commonly used vegetation indices. This showed that the choice of driving data should not be underestimated. Therefore, the driving data for FVC inversion research should be selected with flexibility under different conditions of regions, vegetation type, and seasons [44].

The introduction of multi-dimensional feature set would inevitably be accompanied by the existence of redundant features. In order to avoid redundant features which reduces the computational efficiency and inversion accuracy of the algorithm in the FVC inversion process while also to avoid the limitations of the single feature selection method as well. Among the three feature selection algorithms, the PI-RFE feature selection algorithm constructed in this study had the best dimensionality reduction performance, which retained 15 features as the input data of the machine learning algorithm. Furthermore, the built-in parameters of different machine learning algorithms have a great impact on algorithm performance [13]. The grid search method was used in this study to tune the built-in parameters of the four machine learning algorithms to avoid the uncertainty of artificial selection of reasonable parameter values and to achieve better model accuracy. It is worth mentioning that the regression line seemed to drift farther away from the 1:1 line when the RF algorithm was used for FVC inversion based on the multi-dimensional feature set, which may be due to the over-fitting phenomenon caused by the addition of more functions; however, after the feature selection, there was no such situation. This proved that feature selection and parameter tuning improved the computational efficiency of machine learning algorithms while further improving the FVC inversion accuracy (the R^2^ of RF was higher than 0.90), and also provided better model parameter selection of machine learning algorithms for alpine grassland FVC inversion.

### Evaluation of efficiency and sensitivity for machine learning algorithms

The computational efficiency of machine learning algorithms is considered to be an important evaluation criterion for remote sensing inversion of FVC at high spatial and temporal dimensions [34]. In this study, the training time and prediction time of the four machine learning algorithms were greatly different. For instance, the training and prediction time of the RF algorithm was the shortest, while the SVR algorithm was the longest. These findings indicated that SVR was not suitable for generating long-term serial products [78]. In addition, the sensitivities of different machine learning algorithms to training samples were also different. We found that with the gradual increase in number of training samples, the R^2^ and RMSE of the four algorithms showed a trend of increasing (or decreasing) firstly and then leveling off. However, the inversion accuracy of the RF algorithm and the SVR algorithm exhibited very obvious changes when the sample size was increased from 30 to 120, while the change in inversion accuracy of the BPNN algorithm was relatively stable. Our results suggested that RF and SVR are more sensitive to the sample size than other machine learning algorithms. However, BPNNs were less sensitive to sample size and are an ideal algorithm for FVC inversion with a small sample size. The robustness of the machine learning algorithms was evaluated by analyzing the selection of different training sample sizes. We found that the robustness of the four machine learning algorithms was obviously different when the sample size was small. However, increasing the training sample size obviously improved the stability and differences of this robustness.

## Conclusion

In this study, machine learning algorithms with the best performance among the three commonly used FVC inversion methods were optimized. In addition, a multi-dimensional feature set was constructed, and the dimensionality of the feature set was reduced while quantitatively evaluating the importance of different features in the analysis of FVC of alpine grassland using feature selection algorithms. Finally, optimization algorithms and multi-dimensional features were used to improve the estimation of alpine grassland FVC, and the accuracy was verified by a large amount of measured data. The main conclusions are presented as follows:

1. Using four typical vegetation indices as driving data, it was observed that the machine learning algorithms perform best among the three FVC inversion algorithms. Compared with four typical vegetation indices, the FVC inversion accuracy of the four machine learning algorithms had been improved using the driving data of the multi-dimensional feature set constructed in this study.
2. Topographic factors (DEM, slope, and aspect) and several vegetation indices (VARI, ARVI, SR, and NDVI) played important roles in FVC inversion. The constructed PI-RFE feature selection algorithm had both the best dimensionality reduction effect and the highest accuracy.
3. The combination of feature selection and parameter tuning effectively improved the FVC inversion accuracy of the four machine learning algorithms. The optimized RF algorithm had the highest inversion accuracy and computational efficiency, while the BPNN algorithm was more stable.

In conclusion, the proposed FVC inversion method of alpine grassland is reliable and suitable for operationally producing FVC data. At the same time, it is crucial for the quantitative monitoring of the ecological environment.

## Data Availability

The remotely sensed data and field measured data used in this study is available upon the approval of Dr. Jianjun Chen from College of Geomatics and Geoinformation, Guilin University of Technology, China.

## References

[1] Ahmad F (2012). A review of remote sensing data change detection: comparison of Faisalabad and Multan Districts, Punjab Province, Pakistan. J Geogr Reg Plann.

[2] Altmann A, Tolosi L, Sander O (2010). Permutation importance: a corrected feature importance measure. Bioinformatics.

[3] Bannari A, Asalhi H, Teillet PM. Transformed difference vegetation index (TDVI) for vegetation cover mapping. In: IEEE international geoscience and remote sensing symposium. IEEE; 2002. p. 5. 10.1109/IGARSS.2002.1026867.

[4] Bauer T, Strauss P (2014). A rule-based image analysis approach for calculating residues and vegetation cover under field conditions. CATENA.

[5] Birth GS, McVey GR (1968). Measuring the color of growing turf with a reflectance spectrophotometer. Agron J.

[6] Boegh E, Soegaard H, Broge N (2002). Airborne multispectral data for quantifying leaf area index, nitrogen concentration, and photosynthetic efficiency in agriculture. Remote Sens Environ.

[7] Breiman L (2001). Random forests. Mach Learn.

[8] Bunting EL, Munson SM, Bradford JB (2019). Assessing plant production responses to climate across water-limited regions using Google Earth Engine. Remote Sens Environ.

[9] Castaldi F, Casa R, Pelosi F (2015). Influence of acquisition time and resolution on wheat yield estimation at the field scale from canopy biophysical variables retrieved from SPOT satellite data. Int J Remote Sens.

[10] Chen J, Zhao X, Zhang H (2019). Evaluation of the accuracy of the field quadrat survey of alpine grassland fractional vegetation cover based on the satellite remote sensing pixel scale. ISPRS Int J Geo-Inf.

[11] Chen J, Yi S, Qin Y (2017). The contribution of plateau pika disturbance and erosion on patchy alpine grassland soil on the Qinghai-Tibetan Plateau: implications for grassland restoration. Geoderma.

[12] Chen J, Yi S, Qin Y (2016). Improving estimates of fractional vegetation cover based on UAV in alpine grassland on the Qinghai-Tibetan Plateau. Int J Remote Sens.

[13] Chen J, Sun G, Xing M (2016). A parameter optimization model for geosynchronous SAR sensor in aspects of signal bandwidth and integration time. IEEE Geosci Remote S.

[14] Chen W, Sakai T, Moriya K (2013). Estimation of vegetation coverage in semi-arid sandy land based on multivariate statistical modeling using remote sensing data. Environ Model Assess.

[15] Chen W, Li X, Wang Y (2014). Forested landslide detection using LiDAR data and the random forest algorithm: a case study of the Three Gorges, China. Remote Sens Environ.

[16] Chen Y, Shi P, Li X (2006). A combined approach for estimating vegetation cover in urban/suburban environments from remotely sensed data. Comput Geosc.

[17] Cheng G, Wu T (2007). Responses of permafrost to climate change and their environmental significance, Qinghai-Tibet Plateau. J Geophys Res-Earth..

[18] Cortes C, Vapnik V (1995). Support-vector networks. Mach Learn.

[19] Crippen RE (1990). Calculating the vegetation index faster. Remote Sens Environ.

[20] Deines JM, Kendall AD, Crowley MA (2019). Mapping three decades of annual irrigation across the US High Plains Aquifer using Landsat and Google Earth Engine. Remote Sens Environ.

[21] Demir B, Minello L, Bruzzone L (2014). Definition of effective training sets for supervised classification of remote sensing images by a novel cost-sensitive active learning method. IEEE T Geosci Remote.

[22] Ding Y, Zheng X, Zhao K (2016). Quantifying the impact of NDVIsoil determination methods and NDVIsoil variability on the estimation of fractional vegetation cover in Northeast China. Remote Sens.

[23] Gao L, Wang X, Johnson BA (2020). Remote sensing algorithms for estimation of fractional vegetation cover using pure vegetation index values: a review. ISPRS J Photogramm.

[24] GarcÍA-Haro FJ, Gilabert MA, MeliÁ J (2007). Linear spectral mixture modelling to estimate vegetation amount from optical spectral data. Int J Remote Sens.

[25] García-Haro FJ, Campos-Taberner M, Muñoz-Marí J (2018). Derivation of global vegetation biophysical parameters from EUMETSAT Polar System. ISPRS J Photogramm.

[26] Ge J, Meng B, Liang T (2018). Modeling alpine grassland cover based on MODIS data and support vector machine regression in the headwater region of the Huanghe River, China. Remote Sens Environ.

[27] Georganos S, Grippa T, Vanhuysse S (2017). Less is more: optimizing classification performance through feature selection in a very-high-resolution remote sensing object-based urban application. GISci Remote Sens.

[28] Gitelson AA, Stark R, Grits U (2010). Vegetation and soil lines in visible spectral space: a concept and technique for remote estimation of vegetation fraction. Int J Remote Sens.

[29] Gitelson AA, Kaufman YJ, Merzlyak MN (1996). Use of a green channel in remote sensing of global vegetation from EOS-MODIS. Remote Sens Environ.

[30] Gitelson AA, Merzlyak MN (1998). Remote sensing of chlorophyll concentration in higher plant leaves. Adv Space Res.

[31] Goel NS, Qin W (1994). Influences of canopy architecture on relationships between various vegetation indices and LAI and Fpar: a computer simulation. Int J Remote Sens.

[32] Guerschman JP, Michael JH, Luigi JR (2009). Estimating fractional cover of photosynthetic vegetation, non-photosynthetic vegetation and bare soil in the Australian tropical savanna region upscaling the EO-1 Hyperion and MODIS sensors. Remote Sens Environ.

[33] Guo X, Shao Q, Li Y (2018). Application of UAV remote sensing for a population census of large wild herbivores—taking the headwater region of the yellow river as an example. Remote Sens.

[34] Han M, Liu B (2015). Ensemble of extreme learning machine for remote sensing image classification. Neurocomputing.

[35] Huete A, Didan K, Miura T (2002). Overview of the radiometric and biophysical performance of the MODIS vegetation indices. Remote Sens Environ.

[36] Huete AR (1998). A soil-adjusted vegetation index (SAVI). Remote Sens Environ.

[37] Iizuka K, Kato T, Silsigia S (2019). Estimating and examining the sensitivity of different vegetation indices to fractions of vegetation cover at different scaling grids for early stage acacia plantation forests using a fixed-wing UAS. Remote Sens.

[38] Jia K, Li Y, Liang S (2017). Combining estimation of green vegetation fraction in an arid region from Landsat 7 ETM+ data. Remote Sens.

[39] Jia K, Liang S, Gu X (2016). Fractional vegetation cover estimation algorithm for Chinese GF-1 wide field view data. Remote Sens Environ.

[40] Jia K, Liang S, Liu S (2015). Global land surface fractional vegetation cover estimation using general regression neural networks from MODIS surface reflectance. IEEE T Geosci Remote.

[41] Jiang Z, Huete AR, Didan K (2008). Development of a two-band enhanced vegetation index without a blue band. Remote Sens Environ.

[42] Kaufman YJ, Tanre D (1992). Atmospherically resistant vegetation index (ARVI) for EOS-MODIS. IEEE T Geosci Remote.

[43] Kauth RJ, Thomas GS. The tasselled cap—a graphic description of the spectral temporal development of agricultural crops as seen by LANDSAT. In: Proceedings of the LARS 1976 Symposium of machine processing of remotely-sensed data, West Lafayette. IN: Purdue University. p 4B41–4B51.

[44] Korhonen LH, Packalen P, Rautiainen M (2017). Comparison of Sentinel-2 and Landsat 8 in the estimation of boreal forest canopy cover and leaf area index. Remote Sens Environ.

[45] Kursa MB, Rudnicki WR (2010). Feature Selection with theBorutaPackage. J Stat Softw.

[46] Lehnert LW, Meyer H, Wang Y (2015). Retrieval of grassland plant coverage on the Tibetan Plateau based on a multi-scale, multi-sensor and multi-method approach. Remote Sens Environ.

[47] Li C, Zhu X, Wei Y (2018). Estimating apple tree canopy chlorophyll content based on Sentinel-2A remote sensing imaging. Sci Rep.

[48] Liang S, Ge S, Wan L (2011). Characteristics and causes of vegetation variation in the source regions of the Yellow River, China. Int J Remote Sens.

[49] Liaw A, Wiener M. Classification and regression by randomForest. R News. 2002.

[50] Liu J, Chen J, Qin Q (2020). Patch pattern and ecological risk assessment of alpine grassland in the source region of the Yellow River. Remote Sens.

[51] Ma Y, Wu H, Wang L (2015). Remote sensing big data computing: Challenges and opportunities. Future Gener Comp Sy.

[52] Maimaitijiang M, Ghulam A, Sidike P (2017). Unmanned Aerial System (UAS)-based phenotyping of soybean using multi-sensor data fusion and extreme learning machine. ISPRS J Photogramm.

[53] Melville B, Fisher A, Lucieer A (2019). Ultra-high spatial resolution fractional vegetation cover from unmanned aerial multispectral imagery. Int J Appl Earth Obs.

[54] Meusburger K, Konz N, Schaub M (2010). Soil erosion modelled with USLE and PESERA using QuickBird derived vegetation parameters in an alpine catchment. Int J Appl Earth Obs.

[55] Omer G, Mutanga O, Abdel-Rahman E (2016). Remote Sens.

[56] Otero V, Kerchove RVD, Satyanarayana B (2018). Managing mangrove forests from the sky: forest inventory using field data and Unmanned Aerial Vehicle (UAV) imagery in the Matang Mangrove Forest Reserve, peninsular Malaysia. Forest Ecol Manag.

[57] Patel NN, Angiuli E, Gamba P (2015). Multitemporal settlement and population mapping from Landsat using Google Earth Engine. Int J Appl Earth Obs.

[58] Pinty B, Verstraete MM (1992). GEMI: a non-linear index to monitor global vegetation from satellites. Vegetatio.

[59] Qi J, Chehbouni A, Huete AR (1994). A modified soil adjusted vegetation index. Remote Sens Environ.

[60] Qin Y, Yang D, Gao B (2017). Impacts of climate warming on the frozen ground and eco-hydrology in the Yellow River source region, China. Sci Total Environ.

[61] Ren X, Dong Z, Hu G (2016). A GIS-based assessment of vulnerability to aeolian desertification in the source areas of the Yangtze and Yellow Rivers. Remote Sens.

[62] Rondeaux G, Steven M, Baret F (1996). Optimization of soil-adjusted vegetation indices. Remote Sens Environ.

[63] Roujean J, Breon F (1995). Estimating PAR absorbed by vegetation from bidirectional reflectance measurements. Remote Sens Environ.

[64] Rouse JWJ, Haas RH, Schell JA, et al. Monitoring vegetation systems in the great plains with ERTS. In: third earth resources technology satellite-1 symposium, NASA, WA; 1973; p 309–17.

[65] Rumelhart DE, Hinton GE, Williams RJ (1986). Learning representations by back-propagating errors. Nature.

[66] Song W, Mu X, Ruan G (2017). Estimating fractional vegetation cover and the vegetation index of bare soil and highly dense vegetation with a physically based method. Int J Appl Earth Obs.

[67] Sripada RP, Heiniger RW, White JG (2006). Aerial color infrared photography for determining early in-season nitrogen requirements in corn. Agron J.

[68] Tang L, He M, Li X (2020). Verification of fractional vegetation coverage and NDVI of desert vegetation via UAVRS technology. Remote Sens.

[69] Tao G, Jia K, Zhao X (2019). Generating high spatio-temporal resolution fractional vegetation cover by fusing GF-1 WFV and MODIS data. Remote Sens.

[70] Tu Y, Jia K, Liang S (2018). Fractional vegetation cover estimation in heterogeneous areas by combining a radiative transfer model and a dynamic vegetation model. Int J Digit Earth.

[71] Tucker CJ (1979). Red and photographic infrared linear combinations for monitoring vegetation. Remote Sens Environ.

[72] Verrelst J, Muñoz J, Alonso L (2012). Machine learning regression algorithms for biophysical parameter retrieval: opportunities for Sentinel-2 and -3. Remote Sens Environ.

[73] Wang G, Wang Y, Li Y (2007). Influences of alpine ecosystem responses to climatic change on soil properties on the Qinghai-Tibet Plateau, China. CATENA.

[74] Wang W, Ma X, Nizami SM (2018). Anthropogenic and biophysical factors associated with vegetation restoration in Changting, China. Forests.

[75] Williams M, Bell R, Spadavecchia L (2008). Upscaling leaf area index in an Arctic landscape through multiscale observations. Global Change Biol.

[76] Yao T, Wu F, Ding L (2015). Multispherical interactions and their effects on the Tibetan Plateau’s Earth system: a review of the recent researches. Natl Sci Rev.

[77] Yang K, Ye B, Zhou D (2011). Response of hydrological cycle to recent climate changes in the Tibetan Plateau. Clim Change.

[78] Yang L, Jia K, Liang S (2016). Comparison of four machine learning methods for generating the GLASS fractional vegetation cover product from MODIS data. Remote Sens.

[79] Yang L, Jia K, Liang S (2017). A robust algorithm for estimating surface fractional vegetation cover from Landsat data. Remote Sens.

[80] Yang Z, Willis P, Mueller R. Impact of band-ratio enhanced awifs image on crop classification accuracy. In: Proceedings of the pecora 17 remote sensing symposium. 2008.

[81] Yi S (2016). FragMAP: a tool for long-term and cooperative monitoring and analysis of small-scale habitat fragmentation using an unmanned aerial vehicle. Int J Remote Sens.

[82] Yi S, Zhou Z, Ren S (2011). Effects of permafrost degradation on alpine grassland in a semi-arid basin on the Qinghai-Tibetan Plateau. Environ Res Lett.

[83] Younes N, Joyce KE, Northfield TD (2019). The effects of water depth on estimating Fractional Vegetation Cover in mangrove forests. Int J Appl Earth Obs.

[84] Yu K, Lenz-Wiedemann V, Chen X (2014). Estimating leaf chlorophyll of barley at different growth stages using spectral indices to reduce soil background and canopy structure effects. ISPRS J Photogramm.

[85] Yuan H, Yang G, Li C (2017). Retrieving soybean leaf area index from unmanned aerial vehicle hyperspectral remote sensing: analysis of RF, ANN, and SVM regression models. Remote Sens.

[86] Zabalza J, Ren J, Yang M (2014). Novel Folded-PCA for improved feature extraction and data reduction with hyperspectral imaging and SAR in remote sensing. ISPRS J Photogramm.

[87] Zhang X, Liao C, Li J (2013). Fractional vegetation cover estimation in arid and semi-arid environments using HJ-1 satellite hyperspectral data. Int J Appl Earth Obs.

[88] Zhang Y, Chen L, Wang Y (2015). Research on the contribution of urban land surface moisture to the alleviation effect of urban land surface heat based on Landsat 8 data. Remote Sens.

[89] Zhao W, Li A, Huang Q (2019). An improved method for assessing vegetation cooling service in regulating thermal environment: a case study in Xiamen, China. Ecol Indic.

[90] Zhou Y, Dong J, Xiao X (2019). Continuous monitoring of lake dynamics on the Mongolian Plateau using all available Landsat imagery and Google Earth Engine. Sci Total Environ.

[91] Zhou Y, Li Z, Li J (2018). Glacier mass balance in the Qinghai-Tibet Plateau and its surroundings from the mid-1970s to 2000 based on Hexagon KH-9 and SRTM DEMs. Remote Sens Environ.

[92] Zhou Z, Yi S, Chen J (2018). Responses of alpine grassland to climate warming and permafrost thawing in two basins with different precipitation regimes on the Qinghai-Tibetan Plateaus. Arct Antarct Alp Res.

